# Subtype-Stratified Consensus Gene Signatures: Bridging Tumor Cell Biology, Immune Microenvironment, and Clinical Prognosis in Breast Cancer

**DOI:** 10.3390/ijms27073162

**Published:** 2026-03-31

**Authors:** Xiaoqin Liu, Shang Cai

**Affiliations:** 1 School of Life Sciences, Zhejiang University, Hangzhou 310058, China; liuxiaoqin@westlake.edu.cn; 2 School of Life Sciences, Westlake University, Hangzhou 310024, China; 3 Westlake Laboratory of Life Sciences and Biomedicine, School of Life Sciences, Westlake University, Hangzhou 310024, China; 4 Westlake Disease Modeling Lab, Westlake Laboratory of Life Sciences and Biomedicine, Hangzhou 310024, China

**Keywords:** breast cancer, PAM50 subtype, subtype-specific prognostic gene signature, tumor immune microenvironment (TIME), pathway enrichment

## Abstract

Breast cancer is characterized by profound molecular heterogeneity, which severely limits the clinical utility of universal prognostic tools. To address this gap, we systematically explored transcriptomic profiles in three independent breast cancer cohorts (TCGA, METABRIC, and SCAN-B) via unsupervised clustering. We identified both pan-cancer and PAM50 subtype-specific consensus prognostic gene signatures through log-rank tests and cross-cohort intersection. Single-sample Gene Set Enrichment Analysis (ssGSEA)-derived prognostic scores strongly stratified overall survival across all cohorts, with superior performance over established features (assessed via C-index, time-dependent AUC, NRI, and IDI). Functional enrichment analysis uncovered subtype-specific biological mechanisms: immune-related pathways dominated good-prognostic gene sets in HER2-enriched and Basal-like tumors, while oncogenic pathways characterized poor-prognostic gene sets. Correlation analysis with CIBERSORT-deconvolved immune cell proportions revealed that good-prognostic scores positively correlated with anti-tumor immune cells (CD8+ T cells, M1 macrophages) and negatively with pro-tumor cells (M2 macrophages, Tregs). Independent validation in the Lancet2005 ER+ cohort confirmed that Luminal prognostic gene sets robustly stratified distant relapse-free survival. Collectively, these subtype-specific consensus signatures integrate tumor cell biology and tumor immune microenvironment features, offering robust prognostic tools with potential for future clinical translation.

## 1. Introduction

Breast cancer remains the most prevalent malignancy among women worldwide, with substantial mortality driven by its inherent molecular heterogeneity [1,2,3,4]. The PAM50 classification system has revolutionized breast cancer management by identifying five distinct subtypes (Luminal A, Luminal B, HER2-enriched, Basal-like, and Normal-like) with unique clinical outcomes and therapeutic responses [1,2,3,4]. For example, Luminal A tumors exhibit indolent behavior and sensitivity to endocrine therapy, while Basal-like/triple-negative breast cancer (TNBC) is aggressive and lacks targeted treatment options [5]. Despite this progress, conventional prognostic factors (e.g., tumor size, lymph node status, grade) fail to capture the molecular complexity underlying variable outcomes within subtypes, leading to overtreatment of low-risk patients and undertreatment of high-risk individuals [6,7].

Prognostic gene signatures have emerged as promising complements to clinical factors, but most existing signatures are derived from unstratified cohorts, ignoring subtype-specific biology [8,9]. This “one-size-fits-all” approach limits their generalizability: signatures effective for Luminal tumors often perform poorly in Basal-like or HER2-enriched subtypes. Additionally, many signatures lack functional annotation or validation across independent cohorts, as well as systematic evaluation against clinically established tools (e.g., 70-gene signature [6,10], endoPredict [11,12]), hindering translational application [13]. To address these gaps, there is an urgent need to identify consensus prognostic gene sets that are conserved across patient populations, functionally characterized, and tailored to individual PAM50 subtypes.

Moreover, the tumor immune microenvironment (TIME) has emerged as a critical determinant of breast cancer prognosis and therapy response [9,14,15]. Anti-tumor immune cells (e.g., CD8+ T cells, M1 macrophages) correlate with favorable outcomes, while pro-tumor cells (e.g., M2 macrophages, regulatory T cells) are linked to immune suppression and progression [16]. However, the crosstalk between prognostic gene signatures and the TIME remains poorly understood, particularly at the subtype level. Integrating these two layers of biology could enhance the predictive power of prognostic tools and uncover novel therapeutic targets.

In this study, we aimed to achieve the following: (1) Characterize transcriptomic heterogeneity across PAM50 subtypes in three independent breast cancer cohorts; (2) identify pan-cancer and subtype-specific consensus prognostic gene signatures via cross-cohort validation; (3) evaluate their prognostic performance comprehensively by using multi-dimensional metrics and compare them with established signatures (e.g., gene70, endoPredict); (4) explore their biological functions and correlations with tumor-infiltrating immune cells; (5) conduct independent validation of subtype-specific signatures. We hypothesized that these cross-cohort-derived signatures could enable precise intra-subtype risk stratification, integrate tumor cell biology and TIME features, and outperform clinically established tools in prognostic accuracy and generalizability. These findings offer valuable insights into breast cancer prognostic biology and provide robust, potentially translatable tools for personalized risk assessment and therapy guidance, pending further prospective validation.

## 2. Results

### 2.1. Heterogeneous Gene Expression Profiles Across Breast Cancer Subtypes

Breast cancer is a well-characterized, highly heterogeneous disease, and we first aimed to systematically characterize this heterogeneity at the transcriptomic level. We analyzed gene expression profile data from three independent and well-annotated breast cancer cohorts: The Cancer Genome Atlas (TCGA [2]), Molecular Taxonomy of Breast Cancer International Consortium (METABRIC [1,17,18]), and the SCAN-B (Swedish Cancerome Analysis Network—Breast) study [3,4].

**Figure 1:**
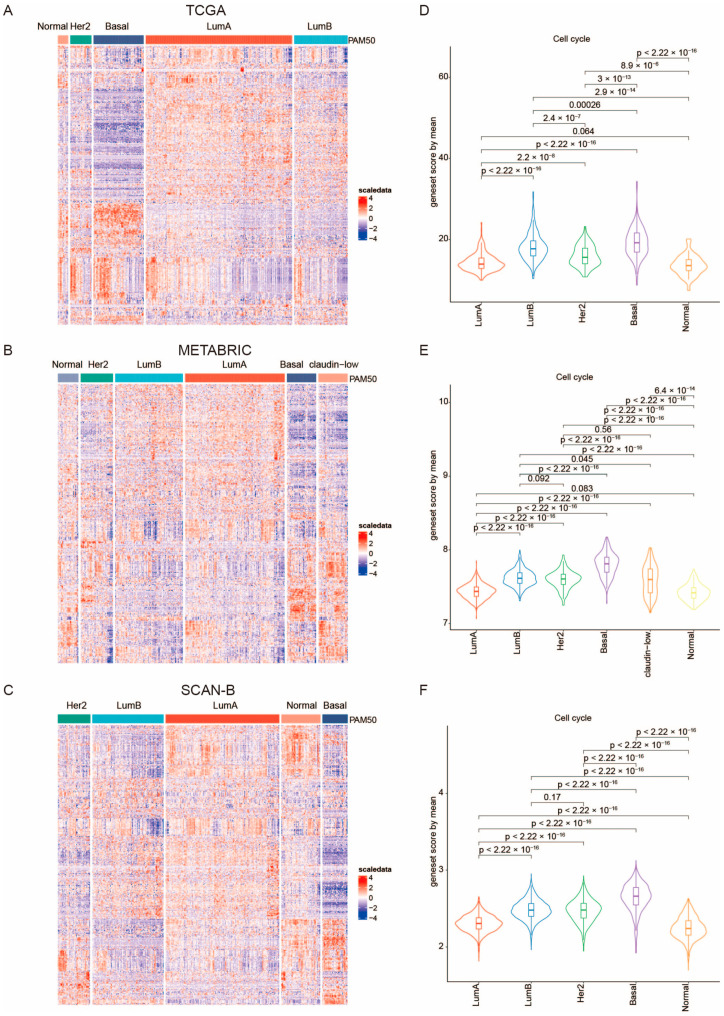
Heterogeneous gene expression profiles across breast cancer subtypes: (**A**–**C**) Unsupervised hierarchical clustering of gene expression profiles in TCGA (**A**), METABRIC (**B**), and SCAN-B (**C**) cohorts, stratified by PAM50 subtypes (Luminal A, Luminal B, HER2-enriched, Basal-like, Normal-like). (**D**–**F**) ssGSEA score difference of cell cycle in TCGA (**D**), METABRIC (**E**), and SCAN-B (**F**).

To examine subtype-specific transcriptional programs, we performed unsupervised hierarchical clustering separately for each PAM50 subtype (Luminal A, Luminal B, HER2-enriched, Basal-like, and Normal-like) within each of the three datasets (Figure 1A–C). This approach allowed us to highlight the consistency of gene expression signatures within each subtype, while simultaneously revealing the stark contrasts between subtypes. Across all three cohorts, we observed that the gene expression profiles of each subtype formed distinct, cohesive clusters, with minimal overlap between different subtypes. For example, Basal-like tumors consistently exhibited a distinct transcriptional profile characterized by high expression of genes associated with proliferation, whereas Luminal A tumors displayed a more differentiated, hormone receptor-driven expression pattern [19] (Figure 1D–F, Supplementary Figure S1A–C).

These observations underscore the substantial molecular diversity inherent in breast cancer and support the notion that this heterogeneity is conserved across different patient populations and experimental platforms. Given the magnitude of these subtype-specific differences, a one-size-fits-all approach to prognostic gene discovery and functional analysis is likely to be insufficient. Therefore, we concluded that it was essential to conduct subsequent analyses both at the overall level and, more critically, separately for each PAM50 subtype to uncover subtype-specific biological insights and prognostic relationships.

### 2.2. Identification of Subtype-Specific Consensus Prognostic Gene Signatures Across Breast Cancer Cohorts

Taking overall survival (OS) as the primary endpoint, we performed single-gene log-rank tests to screen for prognosis-associated genes in each cohort (Supplementary Table S1), identifying 5082, 6744, and 10,178 genes associated with a favorable prognosis in the TCGA, METABRIC, and SCAN-B cohorts, respectively, at the pan-cohort level (Table 1; Supplementary Figure S2A). The intersection of these gene sets across all three cohorts yielded 1267 genes (8.4% of the total), representing a core set of consensus good-prognostic genes (Supplementary Table S2). Similarly, we identified 2737, 7459, and 6518 poor-prognostic genes in each cohort, with a consensus set of 773 genes (6.5% of the total) common to all three (Table 1; Supplementary Figure S2A). For example, the expression of *CASP9*, a gene involved in apoptosis [20,21,22,23] and part of the consensus good-prognostic gene set, was associated with significantly improved overall survival across all cohorts (Supplementary Figure S3A; log-rank *p* = 0.00027 and HR = 0.560 for TCGA, log-rank *p* = 0.00091 and HR = 0.767 for METABRIC, log-rank *p* < 0.0001 and HR = 0.721 for SCAN-B). Conversely, *NF1*, a gene regulating the cytoskeleton [24,25] and part of the consensus poor-prognostic gene set, was associated with significantly worse overall survival (Supplementary Figure S3B; log-rank *p* = 0.016 and HR = 1.796 for TCGA, log-rank *p* < 0.0001 and HR = 1.488 for METABRIC, log-rank *p* < 0.0001 and HR = 1.403 and SCAN-B).

Given the heterogeneity observed, we repeated the analysis to identify subtype-specific prognostic genes, revealing both shared and unique prognostic signatures across the PAM50 subtypes. A total of 229, 163, 124, 23, and 19 consensus good-prognostic genes, as well as 141, 52, 49, 15, and 43 consensus poor-prognostic genes, were identified for Luminal A, Luminal B, HER2-enriched, Basal-like, and Normal-like subtypes, respectively (Table 1; Supplementary Figure S2B–F).

**Figure 2:**
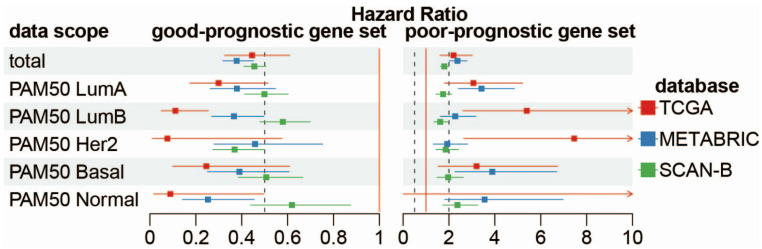
Prognostic effect of subtype-specific consensus gene sets on TCGA, METABRIC, and SCAN-B cohorts. Subtype-specific good-prognostic gene sets showed HR < 1 in three datasets (**left**), while poor-prognostic gene sets showed HR > 1 (**right**). In this figure, red solid vertical lines are reference line at HR = 1 and dashed black vertical lines are guide line at HR = 0.5 or HR = 2. Arrows indicate confidence intervals that extend beyond the axis range.

To examine the clinical relevance of the identified consensus prognostic gene sets (total and subtype-specific good/poor-prognostic genes), we employed ssGSEA [26] to compute the enrichment scores (hereafter referred to as “prognostic scores”) for each gene set in individual samples across the TCGA, METABRIC, and SCAN-B cohorts. This analytical approach converted the collective expression patterns of each gene set into a single quantitative prognostic score for every sample, laying a robust foundation for subsequent survival analyses to evaluate the prognostic value of these gene sets. Survival stratification of samples was initially performed based on ssGSEA-derived prognostic scores via the surv_cutpoint method, with samples stratified into high- and low-score groups for each gene set. Univariate Cox proportional hazards regression analysis was subsequently conducted on these stratified groups. As shown in Figure 2, consistent and significant prognostic trends were observed across all three cohorts: the high-score group of the good-prognostic gene sets exhibited a hazard ratio (HR) < 1, indicating that elevated enrichment scores of good-prognostic gene sets were associated with a lower risk of adverse outcomes, while the high-score group of the poor-prognostic gene sets presented an HR > 1, meaning higher scores of poor-prognostic genes correlated with an increased risk of poor survival. Additionally, to assess the subtype specificity of the identified consensus prognostic gene sets, we compared their prognostic stratification performance across different breast cancer subtypes, and the results (Supplementary Figure S4) support that each subtype-specific gene set exerts an optimal prognostic classification capacity in its corresponding subtype, highlighting the strong subtype selectivity and potential clinical applicability of these gene sets.

To rule out potential biases introduced by the surv_cutpoint stratification method and validate the robustness of the prognostic trends of the gene sets, we further performed two additional analytical strategies for survival analysis. First, we re-stratified the samples into high- and low-score groups using the median value of the prognostic scores as the cut-off; the results (Supplementary Figure S5A) showed that this median-based stratification yielded consistent prognostic trends with the surv_cutpoint method, where the high-score groups of good and poor-prognostic gene sets still presented HR < 1 and HR > 1, respectively, supporting the reliability of the prognostic stratification of the gene sets independent of the cut-off selection method. Second, we treated the continuous prognostic scores as a single variable for univariate Cox regression analysis without any sample stratification; as shown in Supplementary Figure S5B, the continuous scores still maintained the expected prognostic trends across all cohorts, with the good-prognostic gene sets showing a significant HR < 1 and the poor-prognostic gene sets a significant HR > 1, which further verified that the prognostic value of these gene sets is an intrinsic characteristic rather than a result of artificial sample grouping.

Finally, to evaluate the independent prognostic power of the identified total and subtype-specific prognostic gene sets, we conducted multivariate Cox proportional hazards regression analysis by incorporating a series of well-recognized clinical and pathological prognostic covariates—including age, tumor stage, histological grade, lymph node (LN) status, estrogen receptor (ER), progesterone receptor (PR), and human epidermal growth factor receptor 2 (HER2) status, as well as systemic drug treatment status—into the regression model. The results (Supplementary Figure S5C) indicated that even after adjusting for all these confounding clinical factors, the prognostic scores of both the total and subtype-specific good/poor-prognostic gene sets remained significant prognostic factors for overall survival across the three cohorts. This finding supports that the identified consensus prognostic gene sets possess independent and robust prognostic value that is not confounded by traditional clinical and pathological characteristics and can serve as novel complementary prognostic biomarkers for breast cancer, providing additional clinical information for risk stratification and prognostic assessment of breast cancer patients.

Taken together, the ssGSEA-derived prognostic scores for both total and subtype-specific good/poor-prognostic gene sets exhibit robust, cohort-consistent prognostic value for breast cancer survival outcomes, with their stratification and predictive power remaining stable across multiple grouping strategies and independent of traditional clinical-pathological covariates and molecular subtypes. These findings support the potential of the identified prognostic gene sets as biologically meaningful, subtype-selective, and clinically independent prognostic markers for breast cancer, with great potential to complement conventional factors for more precise patient risk stratification and prognostic assessment.

### 2.3. The Total and Subtype-Specific Prognostic Gene Sets Exhibit Superior Prognostic Performance Across Multiple Evaluation Metrics

To comprehensively evaluate the prognostic efficacy of the identified total and subtype-specific good/poor-prognostic gene sets, we systematically assessed their performance using four classic and robust prognostic evaluation metrics, including the Concordance index (C-index), time-dependent area under the curve (time-dependent AUC), Net Reclassification Improvement (NRI), and Integrated Discrimination Improvement (IDI). For NRI and IDI analyses, we compared the prognostic value of our gene sets with two well-recognized and clinically used breast cancer prognostic signatures (70-gene signature, gene70 [6,10], and endoPredict [11,12]) to verify the incremental prognostic value of our identified gene sets over the established biomarkers.

**Figure 3:**
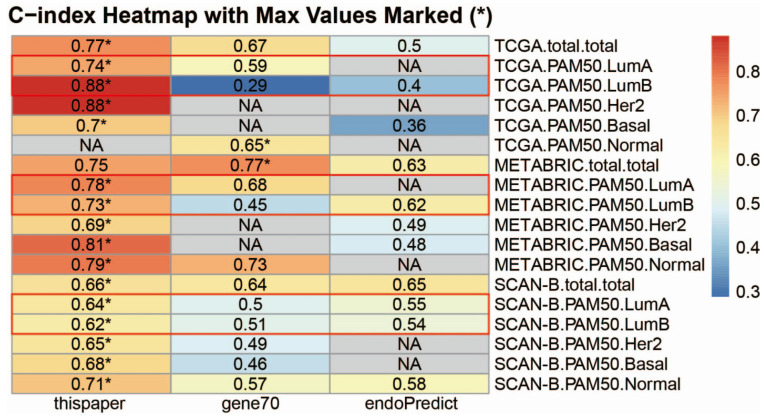
Comparison of C-index among this paper’s prognostic gene sets, gene70, and endoPredict in three cohorts. In most cases, this paper’s C-index showed a higher value than the previous signature. * represents the maximum of each row. Red boxes mark where this study’s signature achieved higher C-index values than gene70 and endoPredict in Luminal subtypes. NA denotes subgroups with insufficient event distribution (only event = 0 or only event = 1), where a valid concordance index could not be computed.

In terms of the C-index, the total and subtype-specific prognostic gene sets consistently showed favorable discriminative ability for survival outcomes across the TCGA, METABRIC, and SCAN-B cohorts (Figure 3; Supplementary Table S3). Notably, our gene sets achieved higher C-index values than both gene70 and endoPredict in the majority of comparisons, particularly within the Luminal subtypes, where these two established signatures are primarily designed to stratify risk [6,10,11,12]. Across all three cohorts, the C-index values of our signature were stably higher than the reference threshold of 0.5 in most PAM50 subtypes, indicating that our prognostic gene sets could effectively distinguish breast cancer patients with different survival risks in both the overall population and subtype-specific subgroups. For example, in the TCGA cohort, our signature reached a C-index of 0.88* in the Luminal B subtype, which was markedly higher than the corresponding C-index values of gene70 (0.29) and endoPredict (0.4). In the METABRIC cohort, our signature outperformed gene70 and endoPredict in the Luminal A, Luminal B, and Basal-like subtypes, while in the SCAN-B cohort, our signature consistently achieved higher C-index values across all PAM50 subtypes, including Normal-like.

The time-dependent AUC analysis further validated the dynamic prognostic predictive power of our gene sets at different follow-up time points (1, 3, and 5 years, Supplementary Table S3). Notably, our gene sets exhibited striking superiority in Luminal A and Luminal B subtypes, with time-dependent AUC values consistently outperforming these two established signatures across all three cohorts. In the Luminal A subtype, our signature showed robust predictive efficacy, with AUC values (0.6 in TCGA, 0.645 in METABRIC, 0.572 in SCAN-B when t = 5) outperforming gene70 (0.502 in TCGA, 0.579 in METABRIC, 0.512 in SCAN-B) and endoPredict (0.5 in TCGA, 0.5 in METABRIC, 0.524 in SCAN-B). For the Luminal B subtype, our gene set achieved an AUC of 0.806 (TCGA, t = 1) and 0.683 (METABRIC, t = 1), which was substantially higher than the corresponding values of gene70 (0.298 in TCGA, 0.507 in METABRIC) and endoPredict (0.468 in TCGA, 0.484 in METABRIC) at the same time points. For the total consensus gene set, our signature showed stable predictive performance across all cohorts and time points (AUC: 0.683 in TCGA at t = 1, 0.688 in METABRIC at t = 3, 0.600 in SCANB at t = 5), while subtype-specific gene sets also support superior efficacy in their corresponding subtypes (AUC = 0.800 in TCGA HER2-enriched at t = 3, 0.955 in TCGA Normal-like at t = 1). These results underscore that our subtype-specific gene sets are tailored to capture the unique molecular characteristics of each subtype, particularly excelling in Luminal A and Luminal B subtypes, where existing clinical signatures are most commonly applied, thereby providing more accurate prognostic prediction for subtype-specific populations.

NRI analysis was performed to quantify the incremental reclassification ability of our gene sets relative to gene70 and endoPredict (Supplementary Table S3). Positive NRI values were observed for the vast majority of gene sets across all three cohorts, indicating improved risk reclassification over these two classic signatures. For the total gene set, NRI values vs. gene70 were 0.294 (TCGA), 0.259 (METABRIC), and 0.199 (SCANB); vs. endoPredict, they were 0.296 (TCGA), 0.365 (METABRIC), and 0.099 (SCANB), with most confidence intervals confirming significant incremental values. In Luminal B, our signature outperformed both gene70 and endoPredict across all three cohorts. In HER2-enriched, it further outperformed these established signatures in METABRIC and SCAN-B. Together, these findings corroborate that our subtype-specific gene sets enable more precise risk reclassification for patients within their corresponding subtypes compared to the previously established prognostic signatures.

Consistent with the NRI results, IDI analysis further verified the integrated discrimination improvement of our gene sets over gene70 and endoPredict (Supplementary Table S3), with positive IDI values observed across almost all gene sets and cohorts, which reflected that our signature could effectively improve the overall discrimination ability for survival outcomes compared with the reference signatures. For the total gene set, the IDI values were stably positive and significant in all three cohorts: 0.033 (95% CI: 0.014–0.059) vs. gene70 and 0.038 (95% CI: 0.013–0.073) vs. endoPredict in TCGA; 0.037 (95% CI: 0.022–0.051) vs. gene70 and 0.046 (95% CI: 0.031–0.062) vs. endoPredict in METABRIC; and 0.002 (95% CI: −0.001–0.006) vs. gene70 and 0.007 (95% CI: 0.004–0.012) vs. endoPredict in SCANB. For the subtype-specific gene sets, distinct patterns of superior prognostic discrimination were observed across different subtypes relative to the reference signatures. In the Luminal A and Luminal B subtypes, our signature consistently outperformed endoPredict across all three cohorts (TCGA, METABRIC, and SCAN-B); compared to gene70, it also showed superior performance in the METABRIC and SCAN-B cohorts. In HER2-enriched and Basal-like subtypes, our signatures further support advantages over both gene70 and endoPredict, with significant performance improvements observed in the METABRIC and SCAN-B cohorts. This subtype-stratified superiority suggests that our subtype-specific gene sets are more tailored to capture the molecular heterogeneity of individual breast cancer subtypes, thereby achieving more accurate prognostic discrimination than gene70 and endoPredict.

Across the multi-metric evaluations (C-index, time-dependent AUC, NRI, and IDI), the identified total and subtype-specific prognostic gene sets have robust, stable, and superior prognostic efficacy for breast cancer survival outcomes across the TCGA, METABRIC, and SCAN-B cohorts. More importantly, compared with the clinically established gene70 and endoPredict signatures, our gene sets show significant incremental prognostic value. These results further imply that our total and subtype-specific prognostic gene sets are more accurate and comprehensive prognostic biomarkers for breast cancer, and can provide more refined risk stratification and prognostic prediction for both the overall breast cancer population and patients with different subtypes.

### 2.4. Functional Enrichment Analysis of Prognostic Genes Across Breast Cancer Subtypes

**Figure 4:**
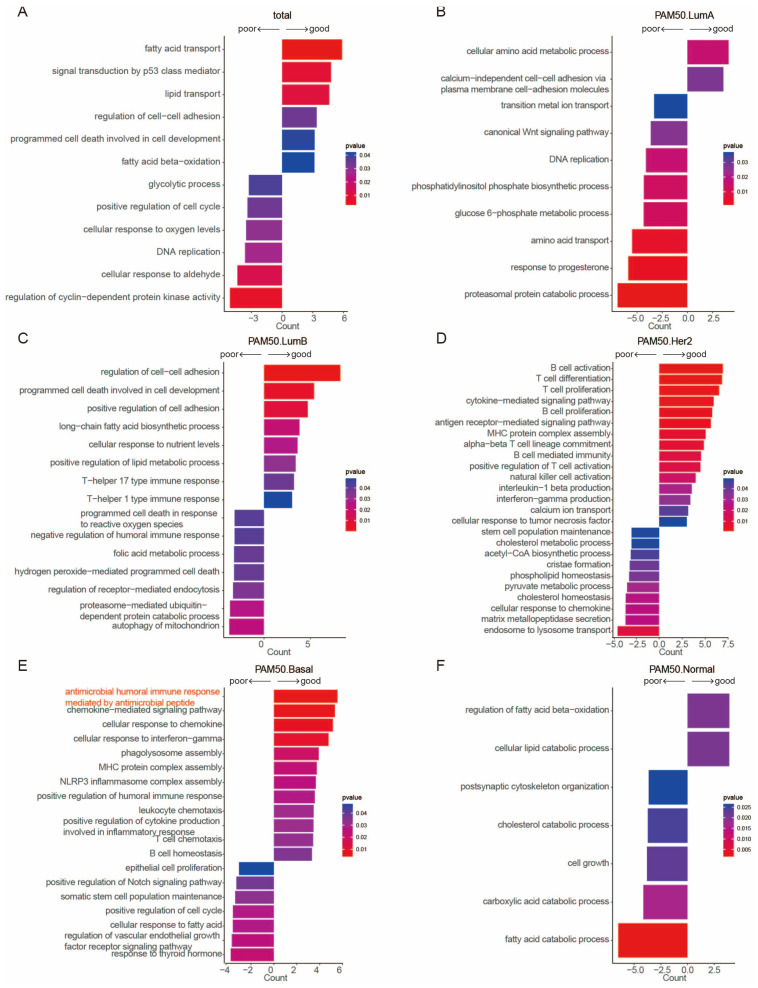
Pathway enrichment analysis of prognostic gene sets: (**A**–**F**) Pathway enrichment bar plots for good (**right**) and poor (**left**)-prognostic gene sets: (**A**) Pan-cancer; (**B**) Luminal A; (**C**) Luminal B; (**D**) HER2-enriched; (**E**) Basal-like; (**F**) Normal-like subtypes. *X*-axis: number of genes in the pathway; *y*-axis: top enriched pathways; color: statistical significance (*p* < 0.05).

Having validated the validity and superior prognostic performance of our gene sets, we next conducted Gene Ontology Biological Process enrichment analysis to elucidate their underlying biological implications and subtype-specific functional divergence (Figure 4A–F; Supplementary Table S4).

In the total cohort, the good-prognostic gene set was enriched in lipid/fatty acid metabolism (e.g., fatty acid transport, beta-oxidation), cell–cell adhesion regulation, and p53-mediated signaling—processes tied to cellular homeostasis and suppressed malignancy [2,27,28,29]—whereas the poor-prognostic gene set converged on hallmarks of aggressive tumorigenesis, including cell cycle progression, DNA replication, glycolysis, and stress responses to oxygen/aldehydes [30,31] (Figure 4A). This pan-cohort pattern is further refined at the subtype level, which uncovers the intrinsic molecular diversity of breast cancer. In Luminal A, good-prognostic gene set correlated with amino acid metabolism and calcium-independent cell adhesion, while poor-prognostic linked to Wnt signaling, DNA replication, progesterone response, and proteasomal catabolism—key drivers of Luminal A proliferation and endocrine resistance [32,33,34,35] (Figure 4B). Luminal B exhibited a unique interplay of metabolism and immunity: good-prognostic genes were enriched in cell adhesion, programmed cell death, long-chain fatty acid metabolism, and Th1/17 immunity reflected a phenotype of structural stability, apoptotic competence, and anti-tumor immune activation [36,37], while poor-prognostic genes were mapped to oxidative stress adaptation, folic acid metabolism, humoral immune suppression, and mitochondrial autophagy, highlighting stress resilience and immune evasion as critical Luminal B progression drivers [38,39,40] (Figure 4C). In HER2-enriched breast cancer, the prognostic dichotomy was defined by immunity versus cellular plasticity: good-prognostic genes were overwhelmingly enriched in adaptive immune processes (B/T-cell activation/proliferation, cytokine signaling, NK cell activation), while poor-prognostic genes clustered in stem cell maintenance, cholesterol/acetyl-CoA metabolism, phospholipid homeostasis, and matrix metallopeptidase secretion—pathways underlying metabolic reprogramming, cancer stem cell persistence, and invasive potential (Figure 4D).

Notably, the Basal-like subtype revealed a previously unreported prognostic mechanism in the context of breast cancer: the good-prognostic gene set was significantly enriched in an antimicrobial peptide-mediated humoral immune response, alongside chemokine signaling, interferon-γ responses, and NLRP3 inflammasome assembly [41,42] (Figure 4E). While antimicrobial peptides are traditionally associated with innate immunity in barrier cells and myeloid lineage cells [43], our findings suggest they may also associate with humoral immune responses in Basal-like tumors, representing a previously unrecognized link between antimicrobial defenses and adaptive anti-tumor immunity that merits further validation. Importantly, this antimicrobial humoral immune pathway effectively stratified Basal-like subtype patients into distinct prognostic subgroups with consistent performance across all three cohorts (Supplementary Figure S6A). Additionally, the antimicrobial peptide DEFB1—a key component of this process—exhibited consistently higher expression levels in the good-prognostic group (Supplementary Figure S6B) and primary Basal tumor-derived cell lines from the CCLE database [44,45,46] (Supplementary Figure S6C), further supporting the pathway’s clinical relevance and potential as a prognostic biomarker for this aggressive breast cancer subtype. Poor-prognostic gene set in Basal-like tumors, by contrast, aligned with epithelial proliferation, Notch signaling, stem cell maintenance, VEGF receptor signaling, and thyroid hormone response—canonical drivers of Basal-like subtype aggressiveness [47,48].

Finally, the Normal-like subtype exhibited a metabolism-centric prognostic signature: good-prognostic genes correlated with balanced fatty acid beta-oxidation and lipid catabolism, while poor-prognostic genes were mapped to dysregulated cholesterol/fatty acid/carboxylic acid catabolism and unconstrained cell growth (Figure 4F), underscoring lipid metabolic rewiring as a core prognostic determinant in this understudied subtype. Collectively, these enrichment analyses imply that our total and subtype-specific prognostic gene sets capture biologically meaningful, subtype-tailored processes and identify novel mechanisms that expand our understanding of breast cancer prognostic biology and offer new avenues for mechanistic validation.

### 2.5. Correlation Analysis Between Tumor-Infiltrating Immune Cell Proportions and Prognostic Gene Scores

**Figure 5:**
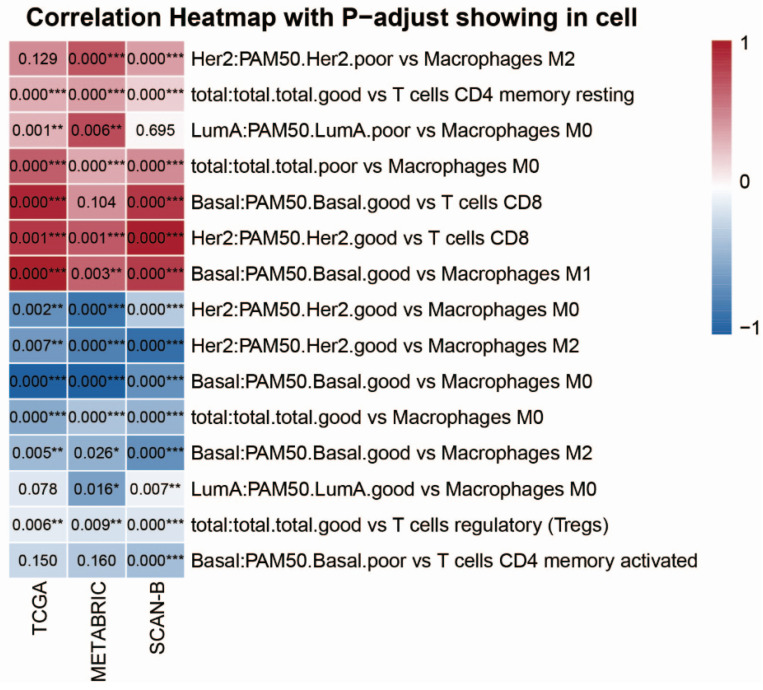
Correlation between prognostic gene set scores and tumor-infiltrating immune cell proportions across breast cancer subtypes. Heatmap showing Pearson’s correlation coefficients between the total/subtype-specific good/poor-prognostic gene set scores and the proportions of tumor-infiltrating immune cell types (estimated by CIBERSORT) in the TCGA, METABRIC, and SCAN-B cohorts. Adjusted *p*-values are displayed within each cell, with significance indicated by asterisks (* *p* < 0.05, ** *p* < 0.01, *** *p* < 0.001).

To dissect the crosstalk between the tumor immune microenvironment and our identified prognostic gene sets, we performed CIBERSORT deconvolution [49] on expression profile data from the TCGA, METABRIC, and SCAN-B cohorts to quantify the proportion of 22 tumor-infiltrating immune cell (TIIC) types (Supplementary Figures S7–S9). We then analyzed the Pearson’s correlation (R) between TIIC proportions and prognostic gene scores (good/poor, subtype-specific), with statistical significance determined by adjusted *p*-values (Figure 5).

Across all cohorts, consistent correlation patterns between the prognostic gene sets and key immune cell populations were identified, which underpin subtype-specific immune-gene crosstalk. In the total breast cancer cohort, the poor-prognostic gene set showed a consistent positive correlation with M0 macrophages, while the good-prognostic gene set was positively correlated with resting CD4 memory T cells and negatively correlated with both M0 macrophages and regulatory T cells (Tregs), supporting that a favorable immune microenvironment is tightly linked to better prognosis in the overall breast cancer population [50,51].

In the Luminal A subtype, the poor-prognostic gene set was positively correlated with M0 macrophages, whereas the good-prognostic gene set showed a consistent negative correlation with M0 macrophages, indicating that M0 macrophage infiltration may be involved in a pro-tumor microenvironment and poor outcomes in this subtype. In the HER2-enriched subtype, the poor-prognostic gene set was positively correlated with M2 macrophages, while the good-prognostic gene set was negatively correlated with both M0 and M2 macrophages and positively correlated with CD8+ T cells—consistent with the notion that a shift toward anti-tumor cytotoxic T-cell infiltration and reduced M2 macrophage polarization is associated with favorable prognosis in HER2-enriched breast cancer [52,53,54,55].

In the Basal-like subtype, the good-prognostic gene set showed consistent positive correlations with CD8+ T cells and M1 macrophages [52,53,56], and negative correlations with M0 and M2 macrophages [50,51], while the poor-prognostic gene set was negatively correlated with activated CD4 memory T cells [57,58]. Notably, the activity of the antimicrobial peptide-mediated humoral immune pathway—identified as a key good-prognostic mechanism in Basal-like tumors—exhibited parallel correlation patterns: it was positively associated with CD8+ T-cell and M1 macrophage proportions and negatively correlated with M0 and M2 macrophage infiltration (Supplementary Figure S10). These findings align with our earlier enrichment analysis, highlighting antimicrobial humoral immunity as a key good-prognostic mechanism in Basal-like tumors, and further supporting that a robust adaptive anti-tumor immune response (elevated CD8+ T cells and M1 macrophages) is a critical determinant of favorable outcomes in this aggressive subtype.

In summary, these consistent correlation patterns—replicated across three independent cohorts—suggest that our prognostic gene sets may be tightly linked to the composition of the tumor immune microenvironment in a subtype-specific manner, with good-prognostic gene sets consistently associated with anti-tumor immune cell populations and poor-prognostic gene sets linked to pro-tumor immune cell infiltration—findings that are consistent with prior research on the prognostic implications of the tumor immune microenvironment in breast cancer.

### 2.6. Prognostic Performance of Luminal A/B Gene Signatures in the Lancet2005 ER+ Breast Cancer Dataset

To empirically validate the clinical utility of our Luminal A/B good/poor-prognostic gene signatures, we performed survival analysis on the independent Lancet2005 ER+ breast cancer cohort [59] by using distant relapse-free survival (DRFS) as the endpoint.

**Figure 6:**
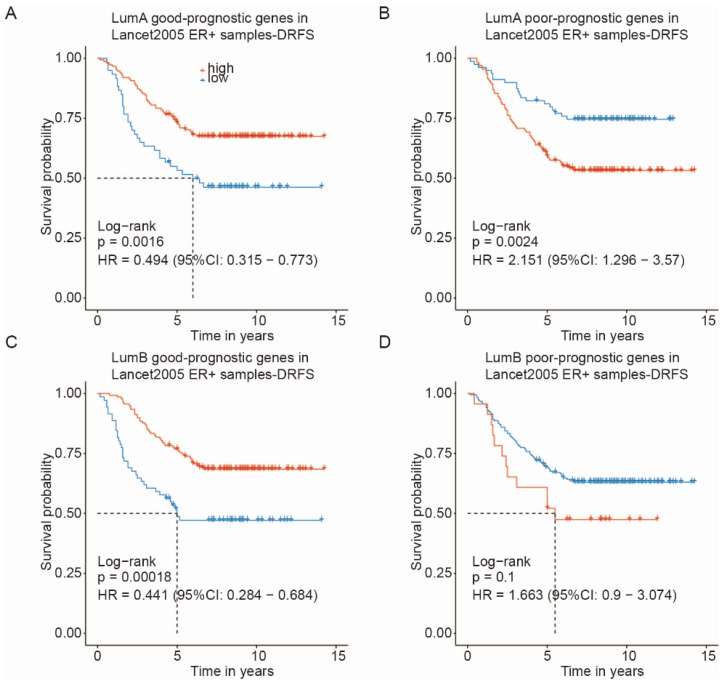
Prognostic performance of Luminal A/B signatures in the Lancet2005 ER+ cohort. (**A**–**D**) KM plots of distant relapse-free survival (DRFS) stratified by Luminal A/B prognostic gene sets in the Lancet2005 ER+ cohort: (**A**) Luminal A good-prognostic gene set; (**B**) Luminal A poor-prognostic gene set; (**C**) Luminal B good-prognostic gene set; (**D**) Luminal B poor-prognostic gene set. Log-rank *p*-values, hazard ratios (HR), and 95% confidence intervals (CI) are displayed in each plot.

For Luminal A signatures, patients with a high expression of the good-prognostic gene set exhibited significantly superior DRFS compared to those with low expression (Log-rank *p* = 0.0016, HR = 0.494, 95% CI: 0.315–0.773; Figure 6A). Conversely, a high expression of the Luminal A poor-prognostic gene set was associated with significantly worse DRFS (Log-rank *p* = 0.0024, HR = 2.151, 95% CI: 1.296–3.57; Figure 6B).

For Luminal B signatures, the good-prognostic gene set, again, stratified patients into distinct prognostic groups, with high expression correlating with significantly better DRFS (Log-rank *p* = 0.00018, HR = 0.441, 95% CI: 0.284–0.684; Figure 6C). While the Luminal B poor-prognostic gene set showed a trend toward worse outcomes (HR = 1.663, 95% CI: 0.9–3.074), this association did not reach statistical significance (Log-rank *p* = 0.1; Figure 6D).

Collectively, these findings in an independent ER+ breast cancer cohort support the potential clinical prognostic utility of our Luminal A and Luminal B gene signatures, particularly the good-prognostic gene sets, which effectively identify patients with favorable distant relapse-free survival outcomes.

## 3. Discussion

Breast cancer’s profound molecular heterogeneity has long undermined the clinical utility of universal prognostic tools, as conventional clinical and pathological factors fail to capture the subtype-specific biological drivers that shape patient outcomes, leading to suboptimal risk stratification and treatment decisions [5,60,61,62]. This study aimed to address this critical gap by developing a subtype-stratified framework for identifying consensus prognostic gene signatures, leveraging three large, independent breast cancer cohorts to define pan-cancer and PAM50 subtype-specific prognostic gene sets, and validating their biological relevance and clinical value through multi-dimensional analyses—including a direct comparison with established clinical biomarkers, functional annotation, TIME correlation, and independent clinical validation. In doing so, we not only provide robust, biologically informed prognostic biomarkers for breast cancer but also establish a mechanistic link between subtype-specific gene expression, tumor cell biology, and immune microenvironment features, laying the groundwork for more precise, personalized risk assessment in clinical practice.

The conserved, distinct transcriptomic profiles of each PAM50 subtype across cohorts support the notion that molecular heterogeneity is a fundamental biological feature of breast cancer [19], not a technical artifact, and directly justified the need for subtype-stratified prognostic research. Our cross-cohort consensus strategy mitigated overfitting and generated the first validated intra-subtype prognostic gene sets for aggressive HER2-enriched and Basal-like subtypes, an unmet clinical need, as prior research has largely focused on Luminal subtypes or pan-cancer signatures with limited utility in non-Luminal disease [6,9,10,11,12]. Converting these gene sets to ssGSEA-derived prognostic scores enabled consistent survival stratification across cohorts, and multivariate Cox analysis confirmed their independent prognostic value, which was unconfounded by traditional clinical covariates.

A head-to-head comparison with clinically established biomarkers (gene70, endoPredict) using four rigorous prognostic metrics demonstrated the superior and broader utility of our subtype-specific signatures. Unlike existing tools, which are restricted to Luminal subtypes and lack efficacy in non-Luminal disease, our signatures exhibited robust prognostic performance across all PAM50 subtypes, with marked improvements in discriminative ability, risk reclassification, and integrated discrimination, even in the Luminal subtypes where gene70 and endoPredict were designed to perform best. This broad subtype applicability addresses a key limitation of current clinical prognostic resources and positions our signature framework as a more comprehensive solution for breast cancer risk assessment.

Functional enrichment analysis uncovered the subtype-specific biological mechanisms underlying the prognostic value of our gene sets, linking signature expression to core oncogenic and homeostatic pathways, and identifying a novel, previously unreported prognostic mechanism in Basal-like breast cancer: an antimicrobial peptide-mediated humoral immune response. This finding advances the current understanding of Basal-like tumor biology and identifies a potential actionable pathway for this aggressive subtype that currently lacks robust prognostic biomarkers. For all subtypes, the functional annotation of prognostic gene sets revealed a consistent dichotomy: good-prognostic genes map to cellular homeostasis and anti-tumor processes, while poor-prognostic genes converge on oncogenic hallmarks such as cell cycle progression, stem cell plasticity, and immune evasion—providing a mechanistic explanation for the clinical prognostic value of the signatures and identifying potential novel therapeutic targets.

Correlation analysis with CIBERSORT-derived TIME profiles further supports that our prognostic signatures integrate both tumor cell-autonomous biology and immune microenvironment features, a key advantage over signatures focused solely on tumor cell gene expression [63,64]. A universal pattern of anti-tumor immune cell association with good-prognostic scores and pro-tumor immune cell association with poor-prognostic scores emerged across cohorts, with subtype-specific refinements that reflect the unique immune landscape of each PAM50 subtype. In the HER2-enriched subtype, we demonstrate that good-prognostic signatures correlate specifically with CD8+ T-cell infiltration and reduced M0/M2 macrophage polarization—a unique immune profile that suggests HER2+ patients with high signature scores may derive greater benefit from anti-HER2 therapy combined with immune checkpoint inhibitors (e.g., anti-PD-1). This subtype-specific correlation was not previously reported and provides a biological rationale for stratifying HER2+ patients into immunotherapy-eligible groups. Most notably, the novel antimicrobial peptide pathway in Basal-like subtypes was tightly linked to favorable anti-tumor immune infiltration (CD8+ T cells, M1 macrophages), indicating that the prognostic value of this pathway may be mediated through modulation of the tumor immune microenvironment and supporting the biological relevance of our signature findings.

Independent validation in the Lancet2005 ER+ cohort supports the clinical translatability of our Luminal subtype signatures, with robust stratification of distant relapse-free survival—a critical clinical endpoint for Luminal disease—despite the signatures being initially trained on overall survival. This cross-endpoint validation supports that our signatures capture the core biological mechanisms underlying both long-term survival and disease recurrence, two key clinical concerns in breast cancer management, and supports their utility in guiding adjuvant therapy decisions for breast cancer patients.

Translating these findings into clinical practice, our subtype-specific consensus prognostic gene sets hold direct translational potential to optimize current treatment strategies by enabling more precise intra-subtype risk stratification. In routine clinical practice, treatment decisions for breast cancer are primarily guided by subtypes: Luminal A subtypes rely on endocrine therapy (with chemotherapy reserved for high-risk cases), Luminal B subtypes receive endocrine therapy combined with chemotherapy (and anti-HER2 therapy if HER2-positive), HER2-enriched subtypes are treated with anti-HER2 therapy plus chemotherapy, and Basal-like subtypes depend on chemotherapy (with immunotherapy added for PD-L1-positive patients) [65,66,67]. However, conventional clinical factors often fail to distinguish risk within each subtype, leading to overtreatment of low-risk patients or undertreatment of high-risk individuals. Our gene sets could address this limitation by further stratifying patients within each subtype: for Luminal A subtypes, low-risk patients (high good-prognostic gene set scores) can safely avoid unnecessary chemotherapy and only receive endocrine therapy, while high-risk patients (high poor-prognostic gene set scores) may benefit from intensified endocrine therapy combined with targeted agents (e.g., CDK4/6 inhibitors); for Basal-like subtypes, low-risk patients can be spared excessive chemotherapy, while high-risk patients require aggressive chemotherapy plus immunotherapy to improve survival outcomes; for HER2-enriched subtypes, risk stratification via our signatures can guide the intensity of chemotherapy combined with anti-HER2 therapy, balancing therapeutic efficacy and treatment-related toxicity. Additionally, our signatures may potentially help resolve intermediate-risk ambiguity (e.g., 1–2 positive lymph nodes) to reduce clinical decision uncertainty and could be compatible with routine RNA-seq/microarray platforms for seamless clinical integration. This refined risk stratification may potentially contribute to reduced overtreatment-related adverse events and improved survival for undertreated high-risk patients, aligning with the core goal of personalized breast cancer care.

While the present study offers meaningful insights into breast cancer prognostic biology, it is not without limitations that should be acknowledged: (1) Reliance on bulk RNA-seq and microarray data masks intratumoral heterogeneity and cannot distinguish tumor cell-specific gene expression from stromal or immune cell expression; (2) the novel antimicrobial peptide-mediated prognostic mechanism in Basal-like subtypes is supported by correlative evidence only, with functional validation needed to confirm causality and therapeutic potential; (3) independent validation of non-Luminal subtype signatures was limited by the scarcity of publicly available cohorts with complete clinical prognostic information. Future research should address these limitations through single-cell RNA-seq to refine tumor-cell-specific prognostic signatures, functional in vitro and in vivo experiments to validate the antimicrobial peptide pathway and other novel prognostic mechanisms, multi-omics integration to enhance signature robustness, and prospective clinical trials to confirm the clinical utility of the signatures.

## 4. Materials and Methods

### 4.1. Data Used

Gene expression data were systematically collected from multiple independent breast cancer (BRCA) cohorts. RNA-seq and clinical data of the TCGA-BRCA cohort (1222 samples total) were downloaded as HTSeq-FPKM files via the GDCquery function in the TCGAbiolinks R package [68,69,70] (v 2.38.0); normal samples were filtered, and only 1109 tumor samples were retained. RNA-seq (FPKM) and clinical data of the SCAN-B cohort (9206 samples) were derived from Staaf et al.’s study [4] (https://data.mendeley.com/datasets/yzxtxn4nmd/4 (accessed on 27 April 2025)). Microarray-based gene expression and clinical data of the METABRIC cohort (1903 samples) were downloaded as the data_expression_median.txt file via the cBioDataPack function in the cBioPortalData R package [71] (v 2.23.2). Microarray and clinical data of Lancet2005 cohort (286 samples) were obtained from Wang et al.’s study [59] (GSE2034).

### 4.2. Subtype-Specific Consensus Prognostic Gene Set Identification and Independence

To identify prognosis-associated gene sets across the entire transcriptome in each cohort, we performed the following steps: (1) Univariate survival analysis: Log-rank tests were conducted for all genes in each of the three independent cohorts (TCGA, METABRIC, and SCAN-B). Analyses were stratified into two categories: (a) all samples in each cohort, and (b) samples stratified by the PAM50 subtype (Luminal A, Luminal B, HER2-enriched, and Basal-like), with each subtype analyzed independently. (2) Significance filtering: Genes were deemed statistically significant if they achieved a false discovery rate (FDR) < 0.05 (Benjamini-Hochberg correction) in the log-rank test. (3) Cross-cohort consensus derivation: Significant genes from the three cohorts were intersected to generate two types of consensus prognostic genes: (i) pan-subtype prognostic genes (good/poor prognosis) associated with survival in the entire breast cancer cohort; (ii) subtype-specific prognostic genes (good/poor prognosis) uniquely linked to survival outcomes for each individual PAM50 subtype.

Patients were separated into two groups based on gene expression or gene set score by using two approaches: the surv_cutpoint and surv_categorize functions in the survminer R package [72] (v 0.5.0), and the median value of ssGSEA-derived prognostic scores as an alternative threshold. ggsurvplot was used to generate Kaplan–Meier (KM) plots for each gene set with corresponding *p*-values. Hazard ratios (HR) and 95% confidence intervals (CI) were obtained from Cox regression analysis. Additionally, we treated the continuous prognostic scores as a single variable for univariate Cox regression analysis without sample stratification. For multivariate Cox proportional hazards regression, we incorporated clinical and pathological covariates (age, tumor stage, histological grade, lymph node status, ER/PR/HER2 status, and systemic treatment) to evaluate the independent prognostic power of the gene sets.

### 4.3. Gene Set Score

To score survival-related prognostic gene sets, we used ssGSEA, which is implemented via the GSVA R package [26] (v 1.44.5), and computes enrichment scores for each gene set in individual samples by ranking all genes and applying a Kolmogorov–Smirnov-like statistic, which is ideal for capturing pathway-level activation without relying on absolute expression thresholds.

### 4.4. C-Index, Time-Dependent AUC, NRI, and IDI Analysis

Samples were first grouped based on ssGSEA scores using the surv_cutpoint method, with overall survival (OS) used as the endpoint. The concordance index (C-index) was calculated using the concordance.index function from the survcomp R package [73,74] (v 1.46.0) to assess the discriminative ability of the prognostic gene sets. Time-dependent area under the curve (AUC) analysis was performed via the timeROC function in the timeROC R package [75] (v 0.4) to evaluate the dynamic prognostic performance of the gene sets at different follow-up time points. For net reclassification improvement (NRI) and integrated discrimination improvement (IDI) analyses, gene70 and endoPredict were set as reference signatures to quantify the incremental prognostic value of our proposed prognostic gene sets over these established signatures. Specifically, NRI and IDI values were derived using the IDI.INF function from the survIDINRI R package (v 1.1-2), enabling a direct comparison of the prognostic performance between our proposed prognostic signatures and the reference signatures (gene70 [6,10] and endoPredict [11,12]).

### 4.5. Function Enrichment Analysis

To investigate the pathways associated with each prognosis-related gene set, we performed Gene Ontology Biological Process enrichment analysis with the clusterProfiler R package [76,77] (v 4.7.1.002). Pathways with a statistical significance threshold of *p* < 0.05 were considered significantly enriched.

### 4.6. CIBERSORT Analysis

To link prognosis-associated gene sets to the tumor immune component, we performed CIBERSORT [49] (v 0.1.0) deconvolution on gene expression profiles for the three datasets separately. We then conducted Pearson’s correlation analysis between the scores of prognosis-associated gene sets and the proportions of tumor-infiltrating immune cell (TIIC) subsets, identifying several significant correlations, with holm adjusted *p* < 0.05 considered significant.

## 5. Conclusions

This study develops a subtype-stratified framework to identify pan-cancer and PAM50 subtype-specific consensus prognostic gene signatures by using three independent breast cancer cohorts. These signatures integrate tumor cell biology and tumor immune microenvironment features, demonstrating robust prognostic performance across multiple metrics (C-index, time-dependent AUC, NRI, and IDI) and outperforming established tools (gene70, endoPredict) across all subtypes—filling the gap of validated prognostic tools for aggressive non-Luminal subtypes. A novel antimicrobial peptide-mediated humoral immune pathway was uncovered as a key prognostic mechanism in Basal-like breast cancer. Independent validation in the Lancet2005 cohort confirms the clinical translatability of Luminal subtype signatures. These findings provide a biologically informed foundation for precise risk stratification and personalized breast cancer care, with potential to reduce over- and undertreatment. Future work will focus on functional validation of novel mechanisms and prospective clinical trials to confirm clinical utility.

## Figures and Tables

**Table 1:** Number of pan-cancer and subtype-specific consensus prognostic gene signatures.

Group	Database	Number of Prognostic Genes (Consensus)			
		Good		Poor	
total	TCGA	5082	(1267)	2737	(773)
	METABRIC	6744		7459	
	SCAN-B	10,178		6518	
Luminal A	TCGA	2110	(229)	2733	(141)
	METABRIC	3917		3836	
	SCAN-B	7699		5612	
Luminal B	TCGA	3336	(163)	1489	(52)
	METABRIC	4190		4700	
	SCAN-B	5798		3908	
HER2-enriched	TCGA	2012	(124)	2085	(49)
	METABRIC	3373		2971	
	SCAN-B	8112		3622	
Basal	TCGA	2828	(23)	2582	(15)
	METABRIC	527		434	
	SCAN-B	7712		3985	
Normal-like	TCGA	421	(19)	627	(43)
	METABRIC	1934		2181	
	SCAN-B	4148		3137	

## Data Availability

Gene expression profiles and clinical data of three cohorts were all from previously published data. All code associated with this study is available on GitHub (https://github.com/Liuxiaoqin95/subtype-specific-consensus-gene-sets).

## Supplementary Materials

The following supporting information can be downloaded at: https://www.mdpi.com/article/10.3390/ijms27073162/s1.
