# Supplementary material for: Subtype-Stratified Consensus Gene Signatures: Bridging Tumor Cell Biology, Immune Microenvironment, and Clinical Prognosis in Breast Cancer
Source: Int J Mol Sci. 2026 Mar 31;27(7):3162. doi: 10.3390/ijms27073162 (PMC13072871; doi:10.3390/ijms27073162)

Supplementary Figure S1

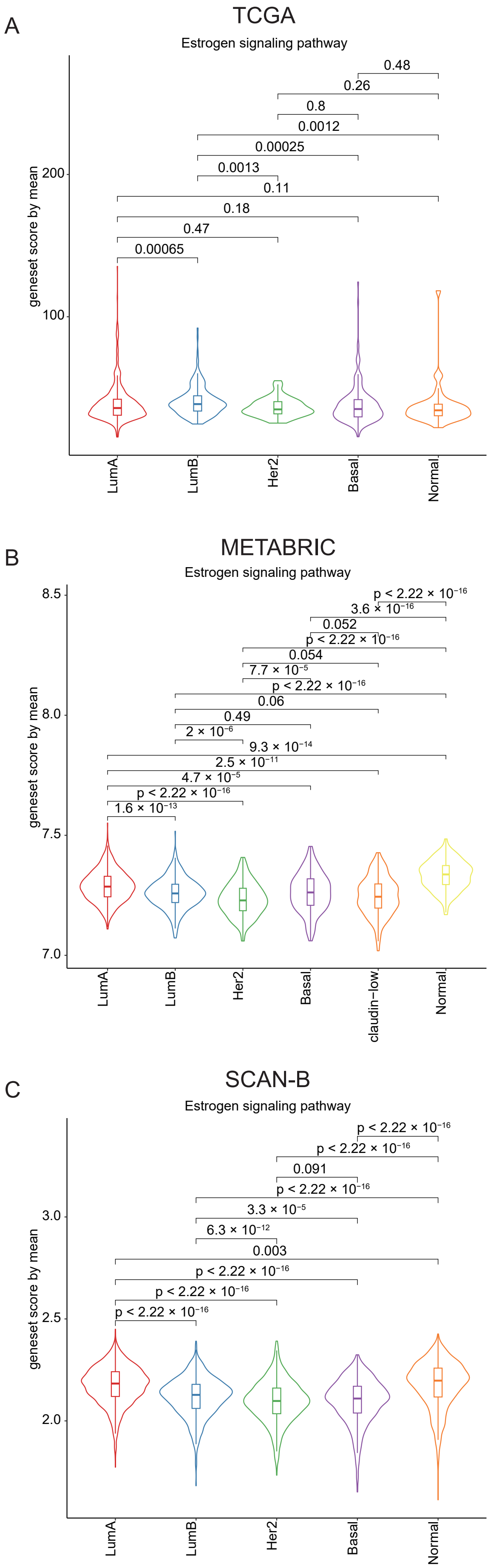

Supplementary Figure S2

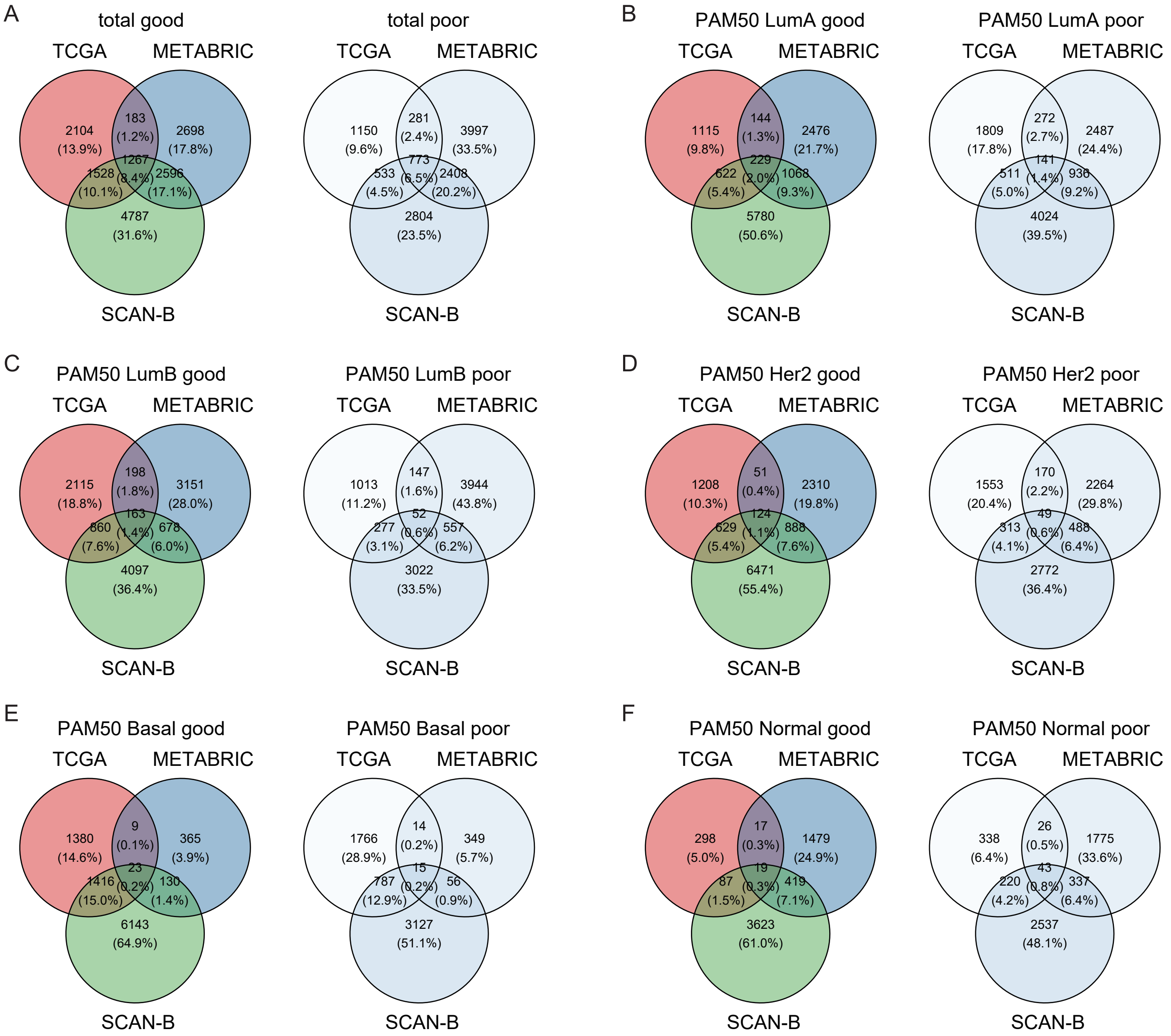

Supplementary Figure S3

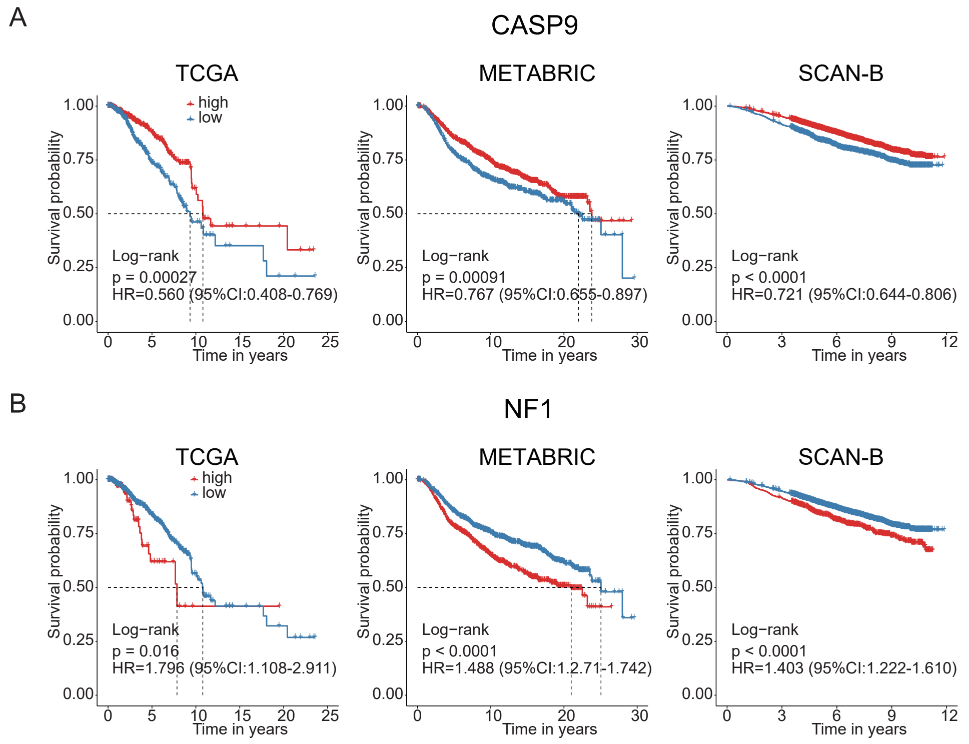

Supplementary Figure S4

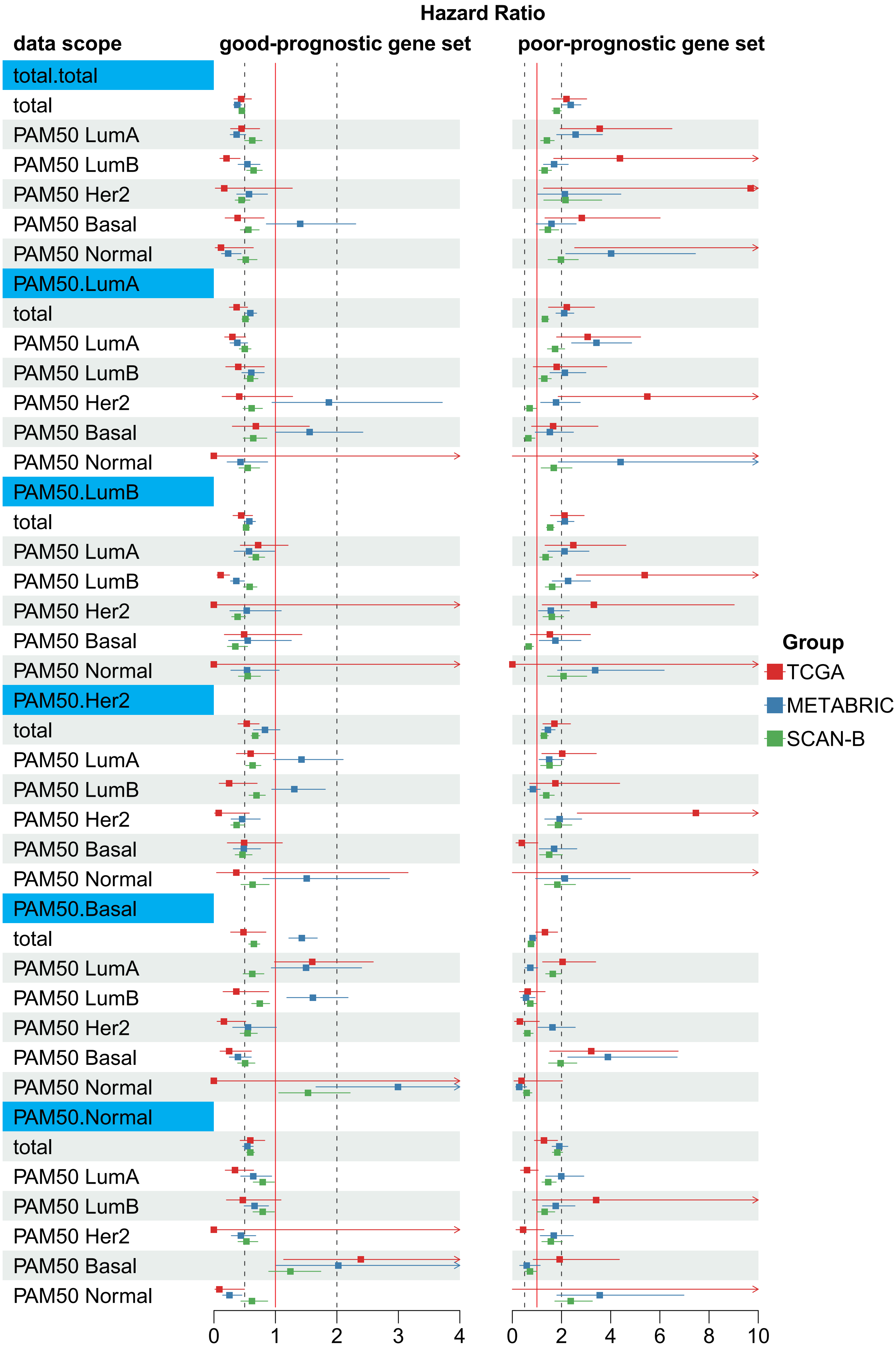

Supplementary Figure S5

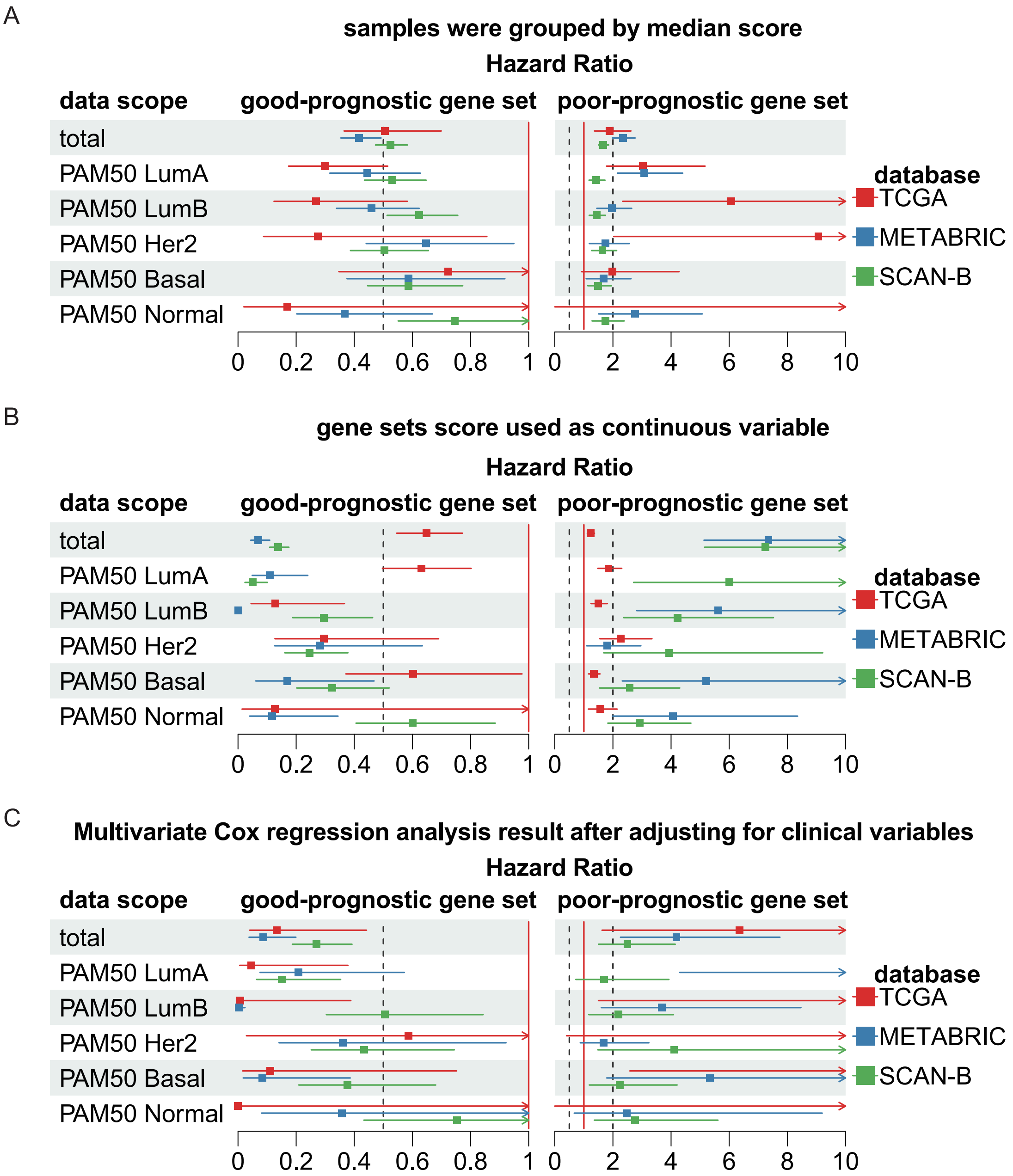

Supplementary Figure S6

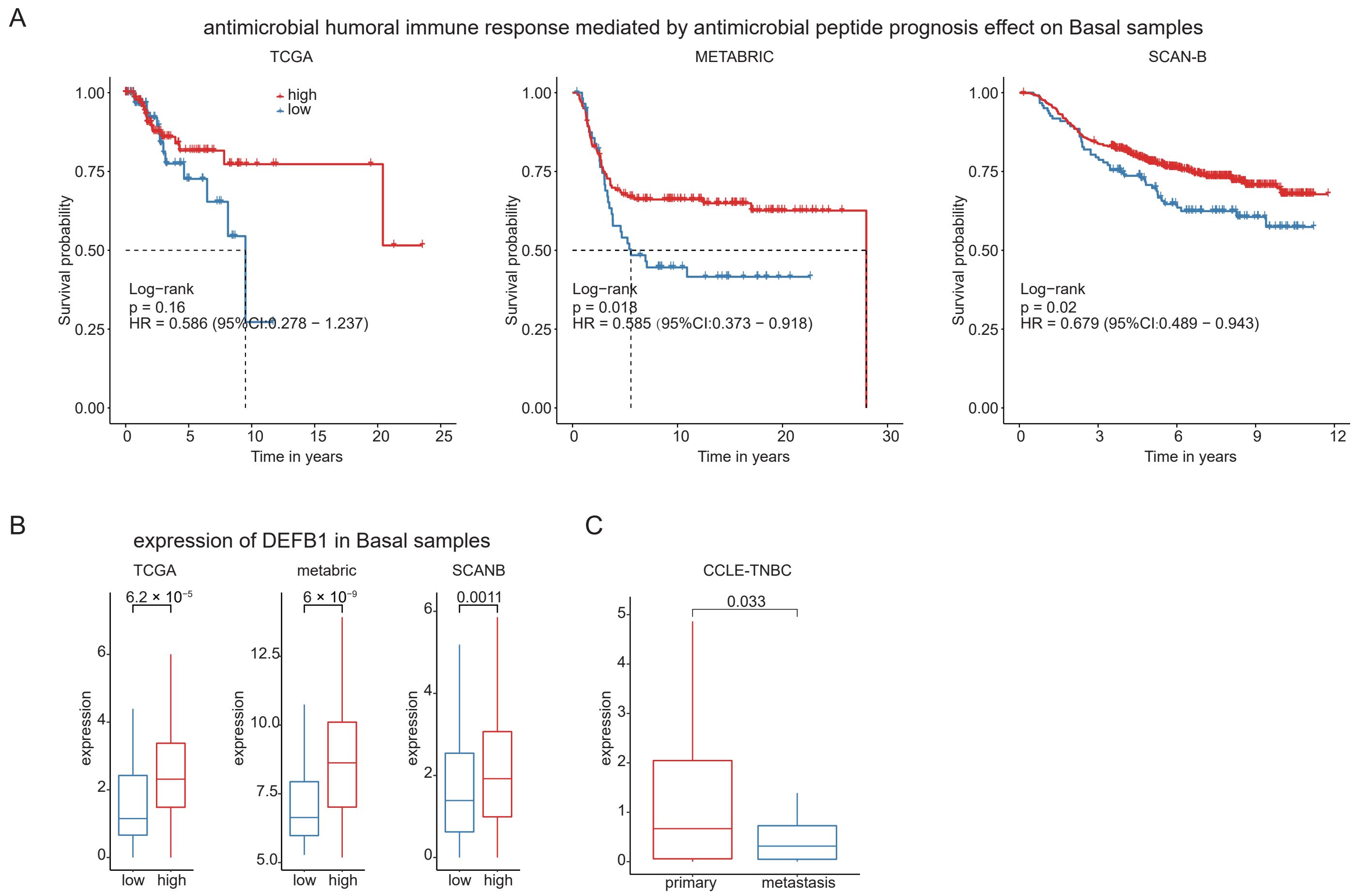

Supplementary Figure S7

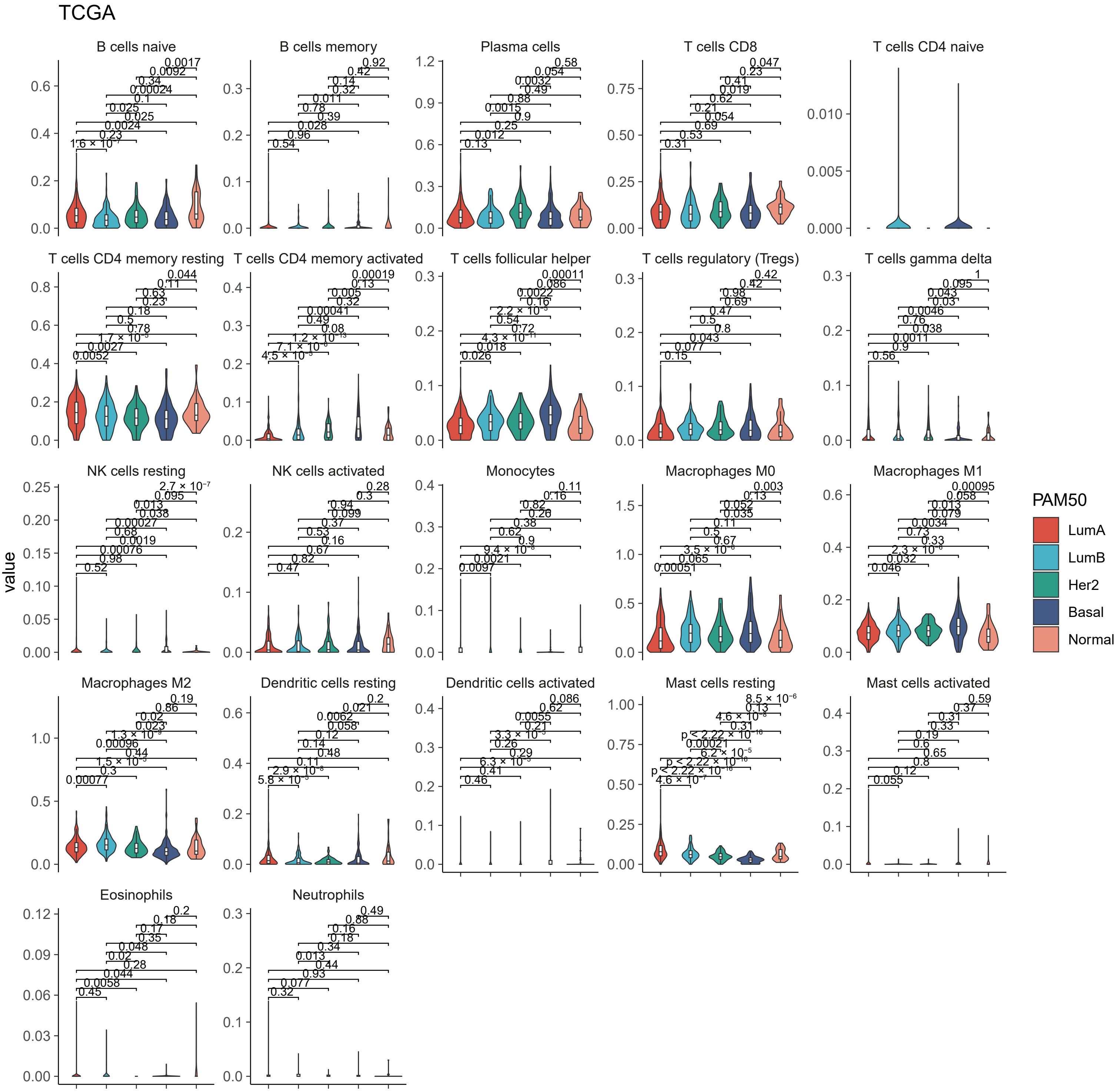

Supplementary Figure S8

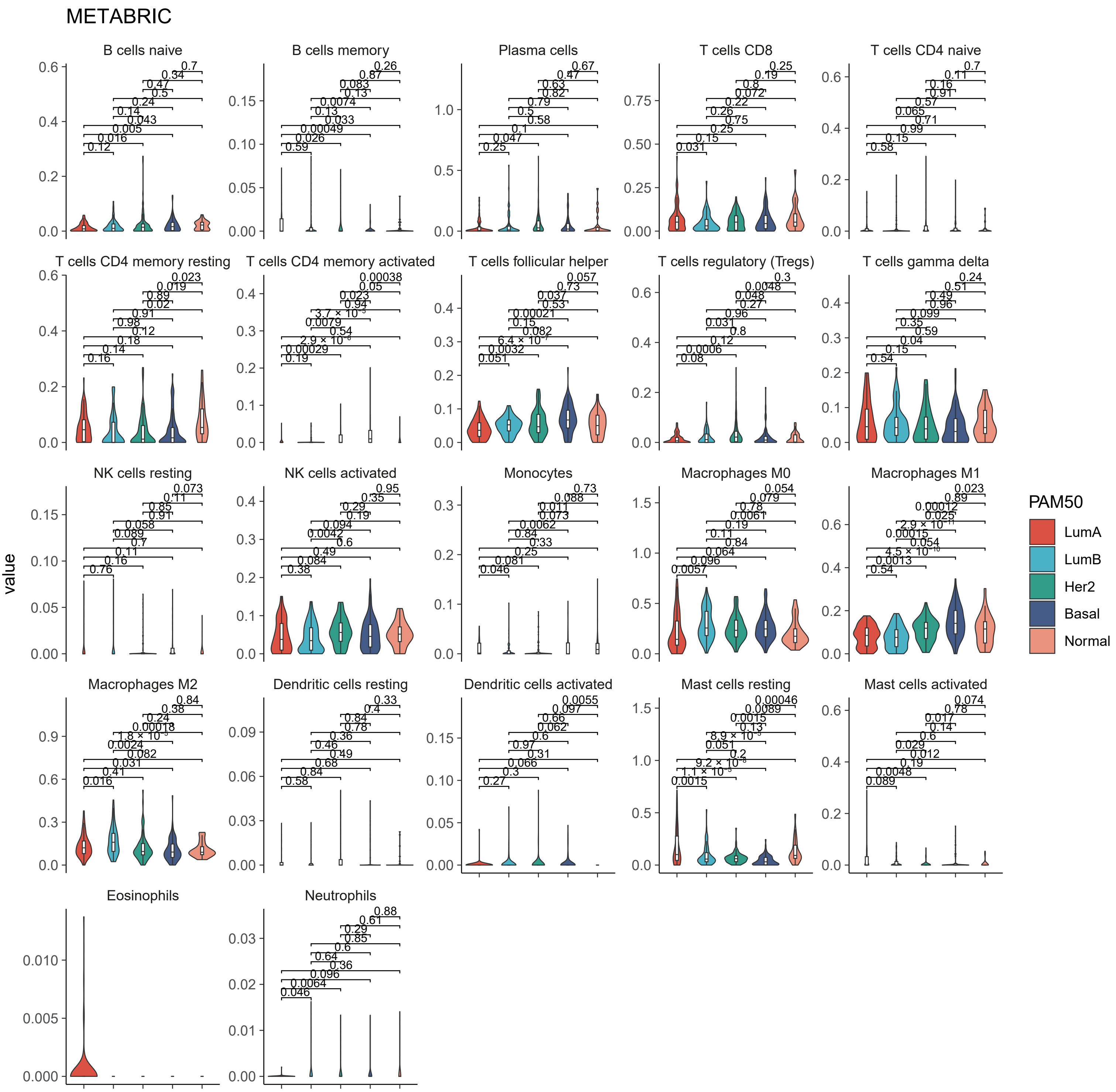

Supplementary Figure S9

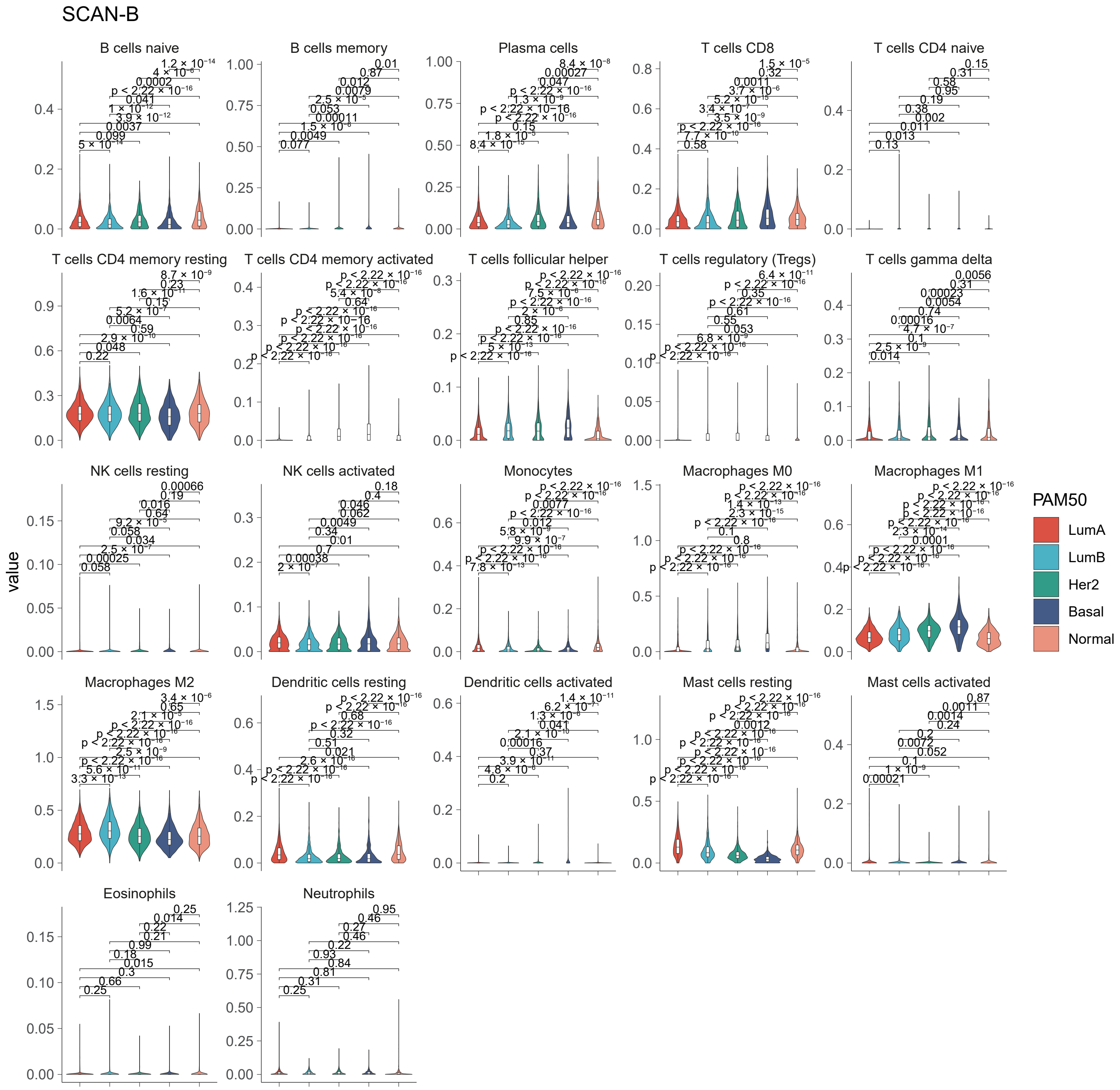

Supplementary Figure S10

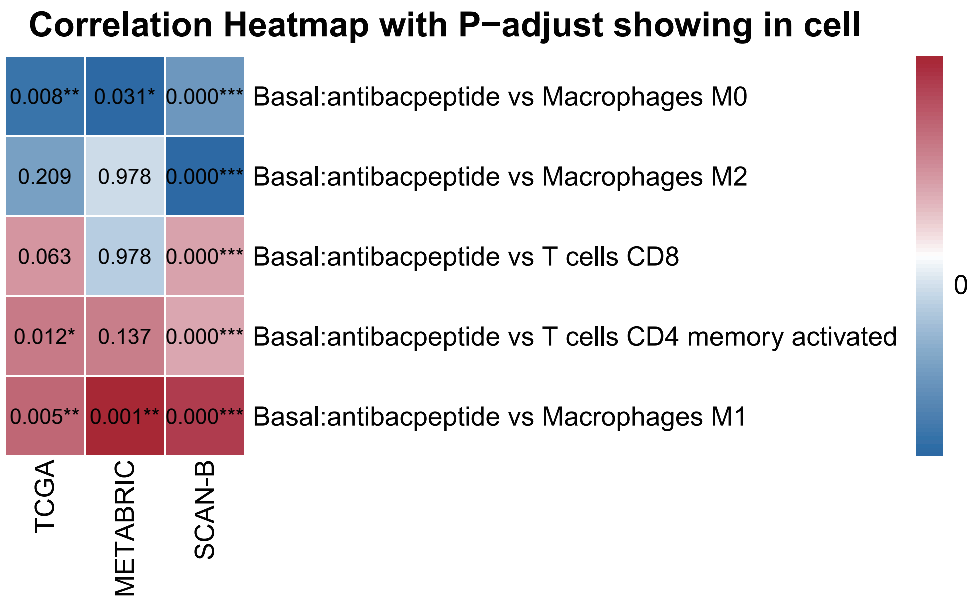

Supplement: Supplementary file 1 [file ijms-27-03162-s001.zip › Supplementary Figures-final.pdf]
