# Supplementary material for: Subtype-Stratified Consensus Gene Signatures: Bridging Tumor Cell Biology, Immune Microenvironment, and Clinical Prognosis in Breast Cancer
Source: Int J Mol Sci. 2026 Mar 31;27(7):3162. doi: 10.3390/ijms27073162 (PMC13072871; doi:10.3390/ijms27073162)
Supplement: Supplementary file 1 [file ijms-27-03162-s001.zip › figure&table legend.pdf]

Supplementary Figure S1. ssGSEA score difference of Estrogen signaling pathway.
(A-C) ssGSEA score difference of Estrogen signaling pathway in TCGA (A), METABRIC (B) and SCAN-B (C).

Supplementary Figure S2. Identification of pan-cancer and subtype-specific consensus prognostic gene signatures.
(A) Venn diagram showing the intersection of good (left) or poor (right)-prognostic genes across TCGA, METABRIC, and SCAN-B cohorts (B–F) Venn diagrams of subtype-specific consensus prognostic genes in Luminal A (B), Luminal B (C), HER2-enriched (D), Basal-like (E), and Normal-like (F) subtypes.

Supplementary Figure S3. Kaplan-Meier plots of two prognostic genes.
(A) KM plots of CASP9 in TCGA, METABRIC, and SCAN-B cohorts. (B) KM plots of NF1 in TCGA, METABRIC, and SCAN-B cohorts.

Supplementary Figure S4. Prognostic effect of subtype-specific consensus gene sets on different breast cancer subtypes in TCGA, METABRIC, and SCAN-B cohorts.
Subtype-specific gene set presented the optimal prognostic classification capacity in its corresponding breast cancer subtype. Subtype-specific good-prognostic gene sets showed HR < 1 in three datasets (left), while poor-prognostic gene sets showed HR > 1 (right). In this figure, red solid vertical lines are reference line at HR=1 and dashed black vertical lines are guide line at HR=0.5 or HR=2. Arrows indicate confidence intervals that extend beyond the axis range.

Supplementary Figure S5. Validation of the robust and independent prognostic value of ssGSEA-derived prognostic scores for the consensus prognostic gene sets across the TCGA, METABRIC and SCAN-B cohorts.
(A) Univariate Cox proportional hazards regression analysis of high- and low-score groups stratified by the median value of ssGSEA prognostic scores for total and subtype-specific good/poor-prognostic gene sets; the consistent prognostic trends (HR<1 for good-prognostic gene sets, HR>1 for poor-prognostic gene sets) across all three cohorts confirm the reliability of the gene set prognostic stratification independent of cut-off selection methods. (B) Univariate Cox proportional hazards regression analysis with ssGSEA prognostic scores treated as a continuous variable for total and subtype-specific good/poor-prognostic gene sets; significant HR<1 for good-prognostic gene sets and HR>1 for poor-prognostic gene sets in all cohorts verify that the prognostic value of the identified gene sets is an intrinsic characteristic rather than an artifact of artificial sample grouping. (C) Multivariate Cox proportional hazards regression analysis of ssGSEA-derived prognostic scores for total and subtype-specific good/poor-prognostic gene sets, adjusted for clinical and pathological covariates including age, tumor stage, histological grade, lymph node (LN) status, ER/PR/HER2 molecular status and systemic drug treatment status; the sustained statistical significance of prognosis scores in all cohorts demonstrates the independent prognostic power of the identified consensus prognostic gene sets, unconfounded by traditional clinical prognostic factors. In this figure, red solid vertical lines are reference line at HR=1 and dashed

black vertical lines are guide line at HR=0.5 or HR=2. Arrows indicate confidence intervals that extend beyond the axis range.

Supplementary Figure S6. Prognostic stratification of the antimicrobial humoral immune pathway in Basal-like breast cancer across three independent cohorts.
(A) Kaplan-Meier (KM) plots showing overall survival (OS) of Basal-like breast cancer patients stratified by the ssGSEA-derived enrichment score of the antimicrobial humoral immune response pathway in TCGA (left), METABRIC (middle), and SCAN-B (right) cohorts. Log-rank p-values, hazard ratios (HR), and 95% confidence intervals (CI) are displayed in each plot. (B) Gene expression difference of DEFB1 between low-score and high-score groups across three cohorts. (C) Gene expression difference of DEFB1 between primary cell line and metastatic cell line derived from Basal type breast cancer from CCLE database.

Supplementary Figure S7. Cell proportion difference among breast cancer subtypes in TCGA.

Supplementary Figure S8. Cell proportion difference among breast cancer subtypes in METABRIC.

Supplementary Figure S9. Cell proportion difference among breast cancer subtypes in SCAN-B.

Supplementary Figure S10. Correlation between antimicrobial peptide pathway and tumor-infiltrating immune cell proportions across breast cancer subtypes.
Heatmap showing Pearson's correlation coefficients pathway scores and the proportions of tumor-infiltrating immune cell types (estimated by CIBERSORT) in the TCGA, METABRIC, and SCAN-B cohorts. Adjusted p-values are displayed within each cell, with significance indicated by asterisks (*p < 0.05, **p < 0.01, ***p < 0.001).

Supplementary Table S1. Log-rank test result on total sample or each breast cancer subtype in three cohorts.

Supplementary Table S2. Intersection of prognostic gene sets among three cohorts.

Supplementary Table S3. Multi-metric prognostic performance evaluation of total and subtype-specific prognostic gene sets across three cohorts.

Supplementary Table S4. Pathway enrichment analysis result for each prognostic gene sets.
